# Altered brain network centrality in patients with cervical spondylotic myelopathy: insights from resting-state fMRI

**DOI:** 10.3389/fneur.2025.1614935

**Published:** 2025-08-13

**Authors:** Kaifu Wu, Hui Zheng, Yan Jiang, Shutong Zhang, Xiang Wang

**Affiliations:** Department of Radiology, The Central Hospital of Wuhan, Tongji Medical College, Huazhong University of Science and Technology, Wuhan, China

**Keywords:** cervical spondylotic myelopathy, resting-state functional magnetic resonance imaging, degree centrality, spinal cord injury, cortical reorganization

## Abstract

**Objective:**

To investigate the characteristics of brain network centrality in patients with cervical spondylotic myelopathy (CSM) by using degree centrality (DC) based on resting-state functional magnetic resonance imaging.

**Methods:**

We recruited 20 patients with CSM, along with 20 healthy controls (HC) who were matched in terms of age, gender, and educational background. The DC method was utilized to evaluate the changed spontaneous brain activities. The relationships between the DC values of different brain regions and the clinical features were analyzed by means of Pearson correlation analysis.

**Results:**

Compared with HC, CSM group showed decreased DC values in the left medial frontal gyrus, middle temporal gyrus and angular gyrus, and increased DC values were found in the left middle occipital gyrus, right supplementary motor area (*p* < 0.05). There was no significant correlation between DC values of abnormal region and clinical function score of CSM patients (*p* > 0.05).

**Conclusion:**

CSM patients have abnormal DC distribution in the whole-brain functional networks, which might be related to cortical reorganization after chronic spinal cord injury.

## 1 Introduction

Cervical spondylotic myelopathy (CSM) refers to spinal cord dysfunction caused by cervical degenerative changes (1–3). It is one of the most common types of spinal cord injury (SCI) and poses a serious threat to the health of middle-aged and elderly people (4). The clinical manifestations of CSM include neck pain, limb numbness, difficulty in performing fine movements, and may eventually lead to paralysis and defecation dysfunction (5–7). In recent years, with the overall aging of society and the accelerated transformation of lifestyles, the incidence of CSM has been increasing, imposing a heavy economic burden to society and families.

CSM has an insidious onset, slow progression, and gradually worsening symptoms, which is a matter of concern for clinical medical workers. Conventional MRI has low sensitivity in detecting CSM, and its imaging changes usually appear in the late stage of the disease, thus possibly missing the optimal treatment opportunity. Previous studies on CSM primarily focused on the spinal cord, and it has been found that cervical spinal cord compression can cause edema, demyelination, damage to fiber microstructure, and restricted diffusion of water molecules in the spinal cord ischemic area (8, 9). Additionally, recent neuroimaging studies have illuminated that CSM not only damages the spinal cord but also triggers extensive atrophy and microstructural changes in the corticospinal tract and distal cerebral cortex (10). As MRI technology advances, its application in CSM has evolved in parallel, transitioning from a mere diagnostic method to a non-invasive tool with the potential to predict neurological prognosis and response to interventions. Researchers like Moxon et al. (11) have noted that the brain functional remodeling in CSM patients shows dynamic changes during recovery process and follows specific spatiotemporal patterns. Resting-state fMRI reflects neuronal functional activities by evaluating changes in blood oxygen saturation and blood flow. It not only provides information about potential neural processing targets but also can be readily applied in clinical settings (12).

Traditional neuroimaging studies have mainly focused on independent component analysis and functional connectivity based on regions of interest (13, 14). However, the former can only present the spatial distribution of networks, while the latter fails to capture the functional connectivity pattern across the entire brain, making it difficult to fully describe the information interaction in core brain regions. Degree centrality is the most direct and widely used analytical method for characterizing the centrality of nodes (brain regions) in resting-state fMRI. It eliminates the need for pre-selecting regions of interest and can directly reflect the interaction between neural clusters and the evolutionary characteristics of network importance (15). This study aims to explore alterations in brain topological attributes in CSM patients through degree centrality analysis of resting-state fMRI, with the hypothesis that CSM leads to distinct changes in key brain hubs.

## 2 Materials and methods

### 2.1 Subjects

A total of 20 right-handed individuals with clinically confirmed CSM were enrolled in this study. The determination of CSM was achieved through clinical and radiological examinations. The CSM group satisfied the exclusion criteria: (1) suffering from psychiatric disorders or major neurologic disorders; (2) having a history of neck surgery previously; (3) being over than 65 years old; (4) having contraindications to MRI examination. Patients with more severe symptoms were more prone to undergoing decompression surgery. The severity of myelopathy was evaluated using the Japanese Orthopaedic Association (JOA) scoring system (16). Meanwhile, 20 matched healthy controls were recruited, all without a history of neurological or psychiatric diseases. The JOA 17-point scale was applied to assess the spinal cord function impairment in CSM patients, mainly including sensory, motor and bladder functions. The lower the score, the more serious the patients’ condition.

All procedures performed in studies involving human participants were in accordance with the ethical standards of the institutional and/or national research committee and with the 1964 Helsinki Declaration and its later amendments or comparable ethical standards. Informed consent was obtained from all individual participants included in the study, and all experimental protocols were approved by the Central Hospital of Wuhan Ethics Committee.

### 2.2 MRI acquisition

A SIEMENS Skyra 3.0 Tesla MRI scanner was utilized to obtain the MR images. In the process of the functional scans, the participants were told to relax with their eyes closed while avoiding falling asleep. Using rubber earplugs to reduce noise and sponge pads to hold the head in place to reduce motion artifacts. Functional MRI datasets were acquired through an echo-planar imaging sequence with the following parameters: repetition time = 3,200 ms, echo time = 30 ms, flip angle = 90°, field of view = 192 × 192 mm^2^, voxel size = 2.0 × 2.0 × 3.0 mm^3^, slice number = 36, slice thickness = 3 mm, totaling 240 time points.

### 2.3 MRI data processing

The preprocessing of raw data was conducted using the Data Processing Assistant for Resting-State fMRI (DPARSF, http://www.restfmri.net), primarily leveraging functions from the Statistical Parametric Mapping software (SPM12, http://www.fil.ion.ucl.ac.uk/spm). The preprocessing steps involved the removal of the initial 10 time points to avoid transient signal instability; slice timing correction, head motion correction (the data were discarded if head motion exceeded 2 mm in translation or 2°in rotation in any direction); normalization into the Montreal Neurological Institute (MNI) space (resampling voxel size of 3 × 3 × 3 mm^3^). Afterwards, the data underwent detrending to eliminate the linear trend of the time course, band-pass filtering in the frequency range of 0.01 ~ 0.08 Hz, and regression of nuisance covariates (including white matter, CSF and the head movement parameters). These steps ensured the refinement and standardization of the resting-state fMRI data for subsequent analyses.

Based on preprocessing, fMRI data were used for DC calculations: Pearson’s correlation analysis of time series was executed between each voxel and every other voxel in the entire brain. A threshold of r > 0.25 was used to derive the adjacency matrix according to previous studies (13). Following this, the individual voxel-wise DC was transformed into a z-score map, and the resulting data underwent spatial smoothing with a 6 mm full-width-at-half-maximum (FWHM) Gaussian kernel. The weighted DC of each voxel was further divided by the global mean DC for standardization.

### 2.4 Statistics analysis

SPSS 25.0 software (IBM Corporation, Armonk, NY, United States) was used to compare the demographic and clinical variables between the two groups. Two-sample t-test was applied for continuous data, and chi-square (χ2) test was used for gender difference. The fMRI data were analyzed using DPABI-Statistical as follows: For voxel-wise DC analysis, a one-sample t-test was conducted to identify DC abnormalities in each group (17). The difference in DC between the CSM and HC groups was evaluated using a mask created by a one-sample t-test statistical map of the two groups. Referring to previous literature (18, 19), inter-group differences were compared using two-sample t-tests (voxel *p* < 0.005, cluster *p* < 0.05, GRF corrected). To explore the potential associations between abnormal DC values and clinical characteristics of CSM, Pearson correlation analysis was performed within the patient group. The threshold for statistical significance was set at *p* = 0.05. *Post hoc* power analysis was performed to assess statistical power where applicable.

## 3 Results

### 3.1 Demographics and clinical characteristics

The demographic and clinical characteristics of the study participants are detailed in Table 1. There were no significant differences between the two groups in terms of age, sex, and education level (all *p* > 0.05). The mean JOA score for CSM patients was 11.50 ± 1.91.

**Table 1:** Demographic and clinical characteristics of CSM patients and HC group.

Characteristic	CSM (*n* = 20)	HC (*n* = 20)	*p*-value
Age (years)	53.50 ± 9.27	49.20 ± 11.06	0.19
Sex (male/female)	9/11	7/13	0.52
Education (years)	11.55 ± 2.24	11.05 ± 2.21	0.48
JOA score	11.50 ± 1.91	NA	-

CSM, Cervical spondylotic myelopathy; HC, Healthy controls; JOA, Japanese Orthopaedic Association.

### 3.2 Imaging differences between groups

**Figure 1:**
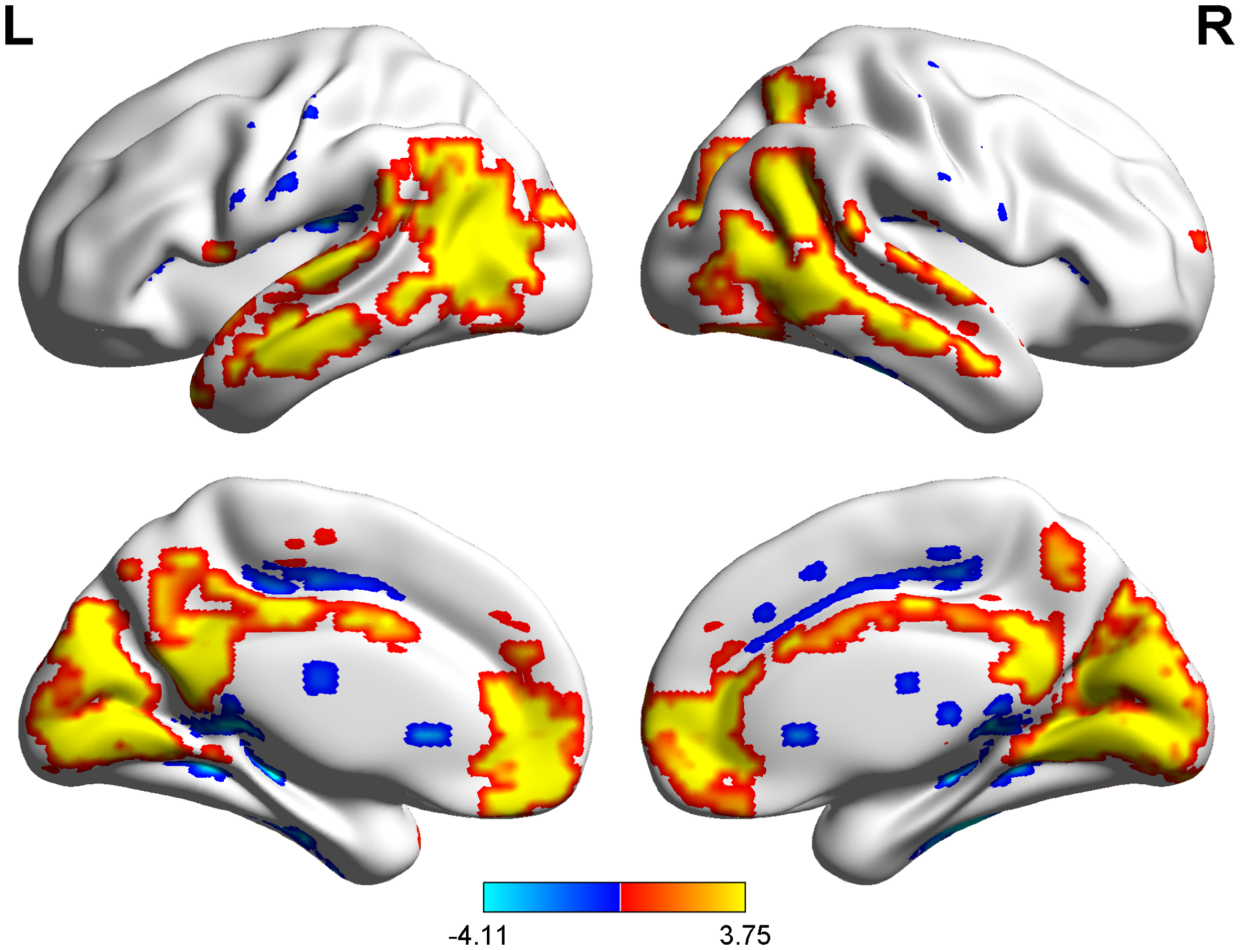
The DC distribution pattern of the HC group.

**Figure 2:**
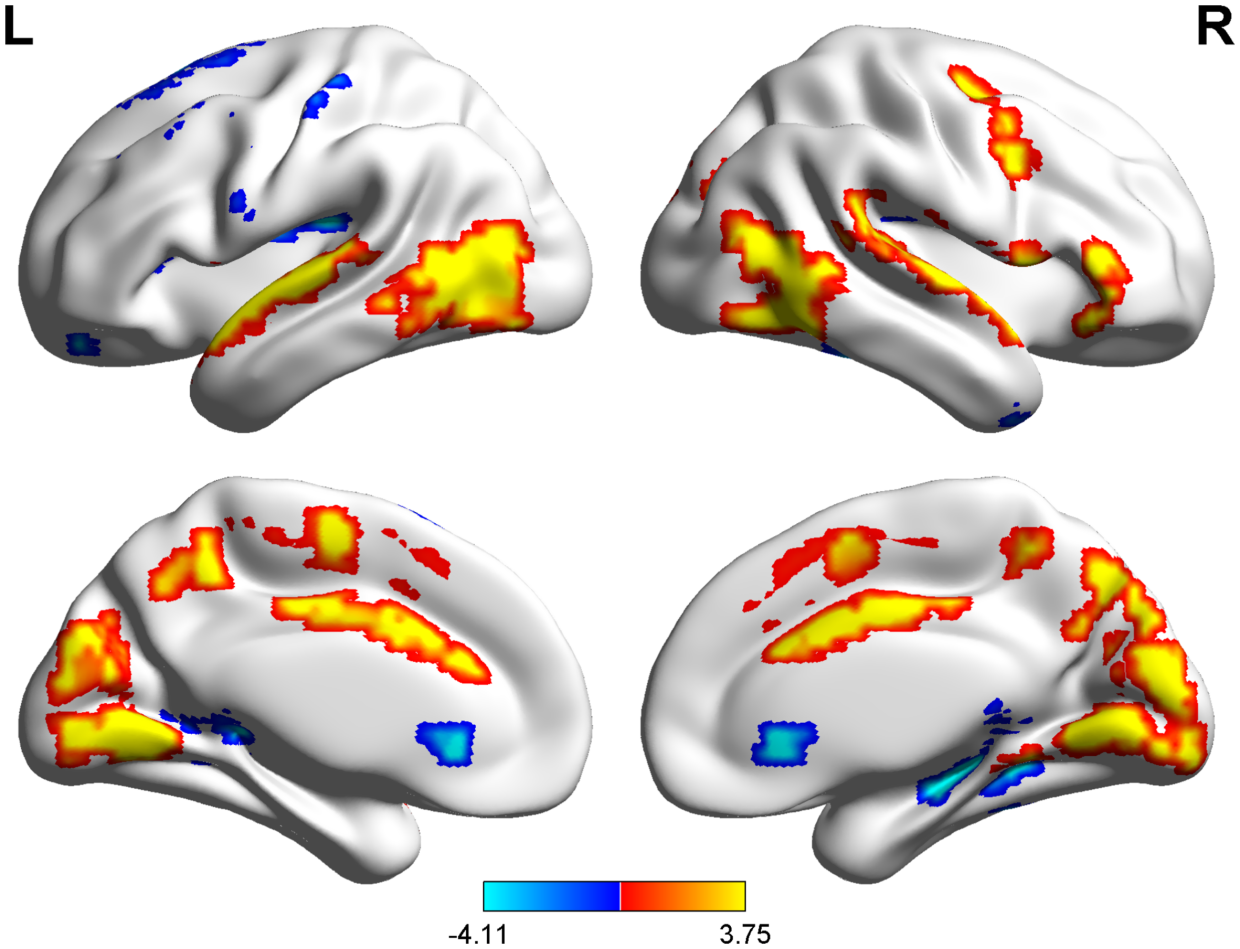
The DC distribution pattern of the CSM group.

**Figure 3:**
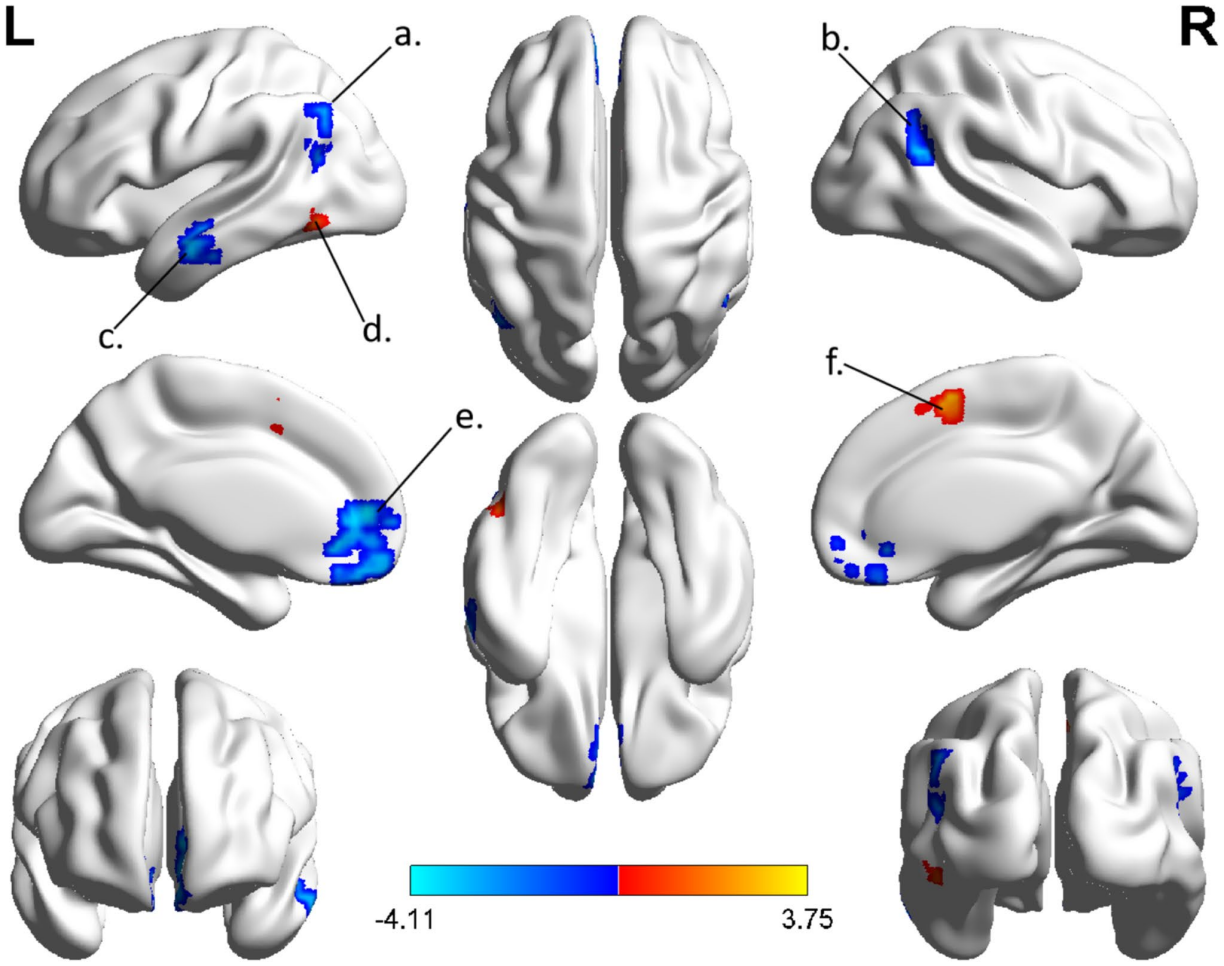
The group differences of DC between CSM patients and HC group. Brain regions with significant differences in DC were identified in the (a) left angular gyrus; (b) right angular gyrus; (c) left middle temporal gyrus; (d) left middle occipital gyrus; (e) left medial frontal gyrus; (f) right supplementary motor area. Hot and cool colors denote higher and lower DC in the CSM patients, respectively.

The spatial distribution patterns of DC variability in HC and CSM patients are shown in Figures 1, 2, respectively. Compared with HC, the CSM group exhibited significantly decreased DC values in regions such as the left medial frontal gyrus, middle temporal gyrus, and angular gyrus. Conversely, increased DC values were observed in regions including the left middle occipital gyrus and right supplementary motor area in CSM patients (Table 2; Figure 3). Notably, no significant correlation was found between DC values in the abnormal regions and the clinical function score of CSM patients (*p* > 0.05).

**Table 2:** Brain regions showed significant differences in DC between CSM patients and HC group.

Condition	Brain regions	MNI coordinate			Voxels	T-value
		X	Y	Z		
CSM < HC	Medial Frontal Gyrus.L	−9	48	6	158	−4.11
CSM < HC	Middle Temporal Gyrus.L	−51	−6	−18	40	−3.54
CSM < HC	Angular Gyrus.L	51	−54	21	41	−3.71
CSM > HC	Middle Occipital Gyrus.L	−54	−69	−6	43	3.75
CSM > HC	Supplementary Motor Area.R	0	3	54	57	3.57

CSM, Cervical spondylotic myelopathy; HC, Healthy controls; MNI, Montreal neurological institute; R, Right; L, Left; GRF-Corrected, voxle level *p* < 0.005, cluster level *p* < 0.05.

## 4 Discussion

The human brain is recognized as one of the most complex homeostatic systems in nature, composed of a vast number of neurons interconnected through synapses (20). These interconnected neuronal clusters synergistically participate in various forms of information processing and cognitive expression within the brain. For a long time, deciphering the working mechanism of the brain and understanding how it regulates behaviors have remained major challenges for neuroscientists (21). Recently, the characterization of brain functional networks has attracted increasing attention in studies on various neuropsychiatric diseases (22). The degree centrality method based on resting-state fMRI can identify key hubs (i.e., core nodes) in the brain. This approach can organically connect different functional regions to construct a complete network matrix, thereby enabling more convenient and comprehensive network connectivity while reducing the loss of metabolic activity information. This study employed the degree centrality method to identify abnormal changes in hub nodes within the brain functional connectivity patterns of patients with CSM. The results further confirm that there is a topological property reorganization in the brain functional network structure of CSM patients.

Previous studies have consistently indicated that in the normal population, the distribution of brain regions with the highest centrality is relatively stable (13), primarily located in the symmetric prefrontal lobe, cingulate gyrus, and temporal lobe—which coincides with the default mode network (DMN) (23). These regions, characterized by high oxygen consumption metabolism and play a core role in the dynamic changes of brain networks. Interestingly, the results of this study are consistent with these previous findings, i.e., the brain regions with the highest centrality in HC group under resting state highly overlap with the DMN. Specific to the CSM patients, we observed a decreased DC in the left medial frontal gyrus, middle temporal gyrus, and right angular gyrus. As an important hub of the DMN, the prefrontal cortex is not only involved in the brain’s regulation of internal and external environmental changes, but also plays a role in emotional integration and episodic memory retrieval (24–26). Previous fMRI studies have found that the left medial prefrontal cortex is associated with self-awareness and is involved in cognitive processing related to self-awareness (27). Zald et al. (28) correlated brain activity in the ventromedial prefrontal cortex with individual differences in negative emotions, while Koenig et al. (29) proposed that this region plays a fundamental role in negative emotion processing, and emotional states can influence behavioral response preparation before movement (30).

Numerous neuroimaging studies have found abnormalities in the angular gyrus across various neuropsychiatric disorders. Song et al. (31) analyzed the functional hierarchy and potential genetic structure of the angular gyrus, revealing that the dominant gradient topology of the angular gyrus is associated with its intrinsic geometric structure. The functional subregions within the angular gyrus that correspond to the classical functional networks (behavioral domains) are hierarchically distributed along the dominant gradient, extending from one end of the DMN (abstract cognition) to the other end of visual and sensorimotor networks (perception and action). The temporal lobe is primarily responsible for processing auditory information and is also associated with memory and emotions. Among its regions, the medial temporal gyrus is not only involved in language-related activities such as semantic memory, word generation, and language processing (32), but also closely linked to emotions and social cognition (33). Moreover, both the posterior part of the middle temporal gyrus and the angular gyrus belong to Wernicke’s area, which is the location of the sensory language center and is mainly responsible for decoding, processing, and storing complex textual information received by the sensory cortex. Up to 60% of patients with spinal cord injury suffer from various types of cognitive deficits (34), including poor attention, impaired visuospatial perception, and learning or memory impairment, which may be associated with the temporal cortex. Minoru and his research team conducted neuropsychological tests on CSM patients with preoperative memory impairment, and the results indicated that this disease may affect cognitive functions, and surgical treatment can ameliorate such effects (35). The findings of this study show that the decreased DC in these core brain regions implies reduced synchronization of neuronal activities and weakened functional connectivity in related brain regions. Such changes may be related to the memory decline and negative emotional responses in CSM patients.

This study also identified brain regions with increased DC, mainly the left middle occipital gyrus and right supplementary motor area (SMA). Damage to the occipital lobe can not only cause visual impairment but can also manifest as memory deficits and motor perception disorders. In recent years, there have been reports on blurred vision symptoms in patients with CSM. Studies by Choe et al. (36) have found that the functional connectivity between the sensorimotor cortex and visual cortex is enhanced in patients with SCI, suggesting that vision-related regions may be involved in compensating for the impaired somatosensory-motor integration function. Patients with SCI, who have not yet recovered the sensory function of the lower body, rely heavily on vision to guide the motor tasks of the lower body. The increased functional connectivity between the sensorimotor and visual networks indicates that the deprivation of sensory input caused by SCI may lead to compensatory functional changes in the patient’s brain, which is beneficial to their rehabilitation. In addition, resting-state fMRI studies have also found increased functional connectivity between the visual cortex and posterior cingulate cortex in CSM patients, indicating that compensatory recruitment occurs in unaffected regions to compensate for the impaired visual cortex (37, 38). The SMA is located on the medial side of the cerebral hemisphere, anterior to the primary motor cortex, and is associated with motor functions. This region is primarily involved in voluntarily initiated movements and plays a role in movement preparation, planning, and sequencing (39, 40). Neural plasticity or cortical reorganization refers to the ability of the nervous system to adapt and restructure its structure, function, and connections in response to internal or external stimuli, a phenomenon that has been confirmed in studies on peripheral or central nervous system injuries (41, 42). A growing body of research has reported evidence of plasticity and compensatory functional reorganization in the brain (43–46), indicating that the impact of CSM on the central nervous system extends beyond the spinal cord (47). For instance, in patients with spinal cord injury, it has been observed that when attempting to move the affected limb, the location of cortical activation expands or shifts (48). It has been reported (49) that compared with healthy individuals, patients who have undergone spinal decompression surgery exhibit an expanded cortical representation for the affected limb. This expansion involves activation of adjacent motor areas, the supplementary motor area, and bilateral cortices. In addition, we attempted to clarify the relationship between the DC values of abnormal brain regions and clinical function score of CSM patients. Unfortunately, we did not find a clear correlation. The possible reasons for this result may be the small sample size of the study or the failure to include clinical indicators such as cognitive assessment, which may have limited the statistical precision in detecting subtle correlations. Furthermore, the variability of disease stages may also have masked potential correlations. It is worth noting that similar negative results have also been reported in previous studies (50–52). For example, no significant correlation was observed between cortical amplitude of low-frequency fluctuations (ALFF) values in the sensorimotor cortex network and JOA scores or disease duration (50).

## 5 Limitations

This study has some limitations. Firstly, the sample size may restrict the precision of the findings; future studies should include larger cohorts and incorporate emotional and cognitive assessments. Secondly, inconsistencies in patients’ disease duration and local spinal cord injury conditions may have impacted the results. Subsequent research will continue to collect relevant cases and postoperative follow-up data, stratifying patients by disease severity and surgical status to reduce heterogeneity. Thirdly, more comprehensive demographic, clinical, and behavioral evaluations will be conducted in the future, combining multimodal MR techniques with machine learning methods to systematically explore the cognitive changes in CSM patients.

## 6 Conclusion

In conclusion, degree centrality analysis revealed abnormal distribution of brain functional hubs in CSM patients, mainly involving key regions of the default-mode network, as well as the visual cortex and supplementary motor area. These findings not only provide imaging evidence for elucidating the neural mechanisms underlying brain remodeling after spinal cord injury, but also offer an important basis for understanding brain-spinal cord interactions and optimizing clinical diagnosis and treatment strategies for CSM.

## Data Availability

The original contributions presented in the study are included in the article/supplementary material, further inquiries can be directed to the corresponding author.
